# Navigating and Advancing Market Access for In Vitro Diagnostics: Understanding the Roles of Key Stakeholders and Policy

**DOI:** 10.1093/ofid/ofaf444

**Published:** 2025-10-22

**Authors:** Brandon K Hill, Andrea M Prinzi, J R Kane, Amalia K Corby, Barbara D Powe

**Affiliations:** US Medical Affairs, bioMérieux, Salt Lake City, Utah, USA; US Medical Affairs, bioMérieux, Salt Lake City, Utah, USA; Public Policy and Advocacy, American Society for Microbiology, Washington, District of Columbia, USA; Public Policy and Advocacy, American Society for Microbiology, Washington, District of Columbia, USA; US Market Access, bioMérieux, Salt Lake City, Utah, USA

**Keywords:** advocacy, health policy, in vitro diagnostics, infectious diseases diagnostics, market access

## Abstract

Rapid and accurate infectious diseases diagnostics are essential to guide antimicrobial stewardship, combat antimicrobial resistance, and improve patient outcomes. However, the availability, adoption, and sustainability of in vitro diagnostics (IVDs) are challenged by high upfront costs, inconsistent coverage and reimbursement policies, and an evolving regulatory landscape. Market access, payor and health policy, and advocacy are pivotal in shaping whether these technologies reach the patient. It is essential to understand the key components of the market access process—regulatory pathways, coverage, reimbursement, evidence generation, and stakeholder engagement—and to recognize how healthcare providers, payors, patients, and policymakers influence diagnostic accessibility. Clinicians, pharmacists, and laboratory professionals must actively participate in advocacy efforts, leveraging clinical insight and evidence to inform regulation, reimbursement, and guideline inclusion. This article presents a practical overview for advancing IVDs through policy and market access engagement, emphasizing the need for coordinated, evidence-based communication with regulatory bodies, payors, and professional societies.

The availability and affordability of infectious diseases (ID) in vitro diagnostics (IVDs) significantly impact antimicrobial stewardship (AMS) and antimicrobial resistance (AMR) mitigation efforts [1]. Rapid and accurate diagnostics are essential to guide targeted therapy, reduce unnecessary antibiotic use, and improve patient outcomes [2]. However, adoption barriers—including high upfront costs, inconsistent payor coverage and reimbursement, and regulatory hurdles—often limit access to these technologies for many laboratories and, ultimately, patients [1, 2]. Market access, healthcare policy, and advocacy efforts are critical yet often underrepresented components of the IVD space. In today's ever-evolving regulatory climate, clinicians and clinical microbiologists must understand these domains. Improved understanding of barriers and champions to access may help ensure that innovative diagnostics are available, affordable, effectively implemented, appropriately utilized, and positioned to improve patient care.

The concept of market access is complex and dynamic and often lacks a consistent definition [3, 4]. Market access encompasses the processes and strategies required to bring an IVD to clinical practice, ensuring its affordability, clinical utility, and integration into healthcare systems. This includes evidence generation, regulatory approval, reimbursement strategies, and advocacy to drive adoption. In their 2024 publication, Shi and colleagues suggested that the ability of patients to access and use a medication, IVD, or other technology is the ultimate outcome of an effective market access strategy [5]. Findings from a scoping literature review within the pharmaceutical industry provide more comprehensive insight into the complexities of market access [3]. Extrapolating from the pharmaceutical industry, market access is defined as a process that begins with the “development and availability of the right products that are proven to be efficacious and disease or condition-specific; specifically produced for the right patients or end users leading to the best clinical outcomes and economic value; delivered at the right time and the right price point in a timely, sustained, and efficient manner; given at the right price (commercially viable or reimbursed price that represents good value); and conducted within the economic, policy, societal, and technological contexts, with the overarching goal of achieving the best patient outcomes and ensuring product profitability” [3].

Health policy refers to the laws, regulations, and guidelines that govern healthcare delivery and access, shaping how IVDs are developed, evaluated, and implemented. Reimbursement refers to the financial models used by public and private payors to cover IVD testing, a crucial factor in test adoption and utilization. Finally, government affairs involve the engagement of stakeholders—including professional societies, policymakers, and regulatory bodies—to advocate for policies that promote innovation and access to diagnostic technologies. Many professional and community organizations have engaged in collaborative advocacy efforts to highlight potential challenges and proposed policy reforms to address them [6, 7]. Clinicians and laboratory staff must increase their engagement in policy and advocacy discussions to ensure ongoing innovation and support for IVDs to achieve widespread adoption and access. Optimal support of innovation and test adoption or access may be achieved by strengthening relationships with industry partners, regulatory bodies, government agencies, professional societies, and patient advocacy groups. Active participation in such efforts could help shape policies that improve patient outcomes and advance diagnostic stewardship for ID.

Understanding how market access is shaped enables clinicians to become champions for change. Advocacy efforts—from participation in professional society initiatives to direct engagement with policymakers—can influence reimbursement policies and drive regulatory adaptations. This article aims to present a clear and practical overview of market access and health policy, empowering ID clinicians and clinical microbiologists to advocate for improved innovation and access to IVDs.

## KEY STAKEHOLDERS IN THE MARKET ACCESS PROCESS

### Healthcare Payors

Government and private payors are major stakeholders in the United States (US) healthcare system and the market access landscape as they shape patient access and coverage decisions and influence reimbursement. IVD manufacturers must demonstrate that an IVD is safe, effective, and meets established regulatory standards [8]. However, to ensure successful market access, manufacturers must demonstrate the product's clinical and economic value during pricing and reimbursement negotiations [4]. In the US, Medicare is the largest payor for healthcare services [9]. Methodologies used by the Centers for Medicare and Medicaid Services (CMS) to determine payment rates may vary by setting and have different statutory and regulatory requirements [9, 10]. A National Coverage Determination (NCDs) is a decision by CMS on whether a service or item is covered and reimbursed by Medicare [11]. The NCD process is evidence-based and relies on scientific evidence and medical reviews, with opportunities for public comment [11]. The CMS uses a network of 12 regional, multistate, Medicare Administrative Contractors (MACs) to serve as the primary operational contact between the Medicare fee-for-service program (Medicare Parts A and B) and the healthcare providers enrolled in the program [12]. Within the MAC network, a Local Coverage Determination may be developed if an NCD does not exist or there is a need to further define an existing NCD [13].

Payment rates and policies developed by CMS frequently form the foundation for private insurance coverage and reimbursement decisions. The predominant form of health coverage in the US is, and historically has been, private health insurance through an employer or individual [14]. Covered benefits are the health services (eg, physician visits, surgeries) and items (eg, prescription drugs) that a plan will cover, either fully or partially, for their enrollees. A plan's coverage of a given benefit generally will depend on the benefit being deemed medically necessary and not those considered experimental or investigational [14, 15]. Covered benefits may differ by plan. Decisions on medical necessity may be based on factors like scientific evidence, established medical guidelines, the potential health benefit of the test, and whether relevant regulatory bodies have approved it [14]. Trosman and colleagues suggest that in the US multipayor system, insurance coverage not only varies by payor but also by what is covered as well as when or if individual payors decide to provide coverage [15]. For example, one payor's policy may cover the IVD test in an inpatient setting but not in an outpatient setting, or the policy may define specific diagnoses or symptomatology as criteria for defining medical necessity for using the test. A payor's coverage decision may influence a provider's decision to order the test for their patients. While achieving standardization of IVD coverage among private payors may not be achievable, Wempe et al recommend that payors develop a minimum set of performance characteristics needed to support coverage determinations for IVDs to ensure access and accurate results and limit exposure to ineffective or unneeded testing or treatments [16]. These performance metrics should also inform the manufacture of types of evidence preferred and other strategies that would lead to favorable coverage decisions.

Government payors (eg, CMS), private payors, and hospitals use medical coding and billing to obtain reimbursement. Current Procedural Terminology (CPT) codes are clinically focused and utilize common standards so that a diverse set of users can have common understanding of the procedure across healthcare settings [17]. The CPT terminology is the most widely accepted and used in the US to report medical, surgical, radiology, laboratory, anesthesiology, genomic sequencing, evaluation, and management. CPT coding facilitates payment, so procedures and devices are not reimbursed at the hospital or healthcare provider level without a proper code [17, 18]. However, the payor's policy and coverage guidelines determine if or to what extent the payor reimburses the service. Simply stated, a CPT code is required as a part of the billing process; however, having a CPT code will not guarantee coverage of the service by the payor.

This manuscript does not include an exhaustive discussion of the CPT codes and the coding process. However, the types of CPT codes (Table 1) are described as Category I, II, III, or Proprietary Laboratory Analyses codes [17]. The IVD manufacturer is responsible for establishing the appropriate CPT code for their product. Options include creating a new code, obtaining an add-on code for additional payments on an existing procedure, or grouping the product within an existing CPT code by following the application process published by the American Medical Association's CPT® Editorial Panel [17–19].

**Table 1.** Overview of Current Procedure Terminology Codes

Type of CPT Code	Brief Description
Category I	Descriptors correspond to a procedure or service. Codes range from 00100 to 99499 and are generally ordered into subcategories based on procedure/service type and anatomy.
Category II	These alphanumeric tracking codes are supplemental codes used for performance measurement. Using them is optional and not required for correct coding.
Category III	These are temporary alphanumeric codes for new and developing technology, procedures, and services. They were created for data collection, assessment, and in some instances, payment of new services and procedures that currently do not meet the criteria for a Category I code.
Proprietary Laboratory Analyses	These codes describe proprietary clinical laboratory analyses and can be either provided by a single (“sole source”) laboratory or licensed or marketed to multiple providing laboratories that are cleared or approved by the US Food and Drug Administration.

Adapted from: American Medical Association [17].

Abbreviation: CPT, Current Procedural Terminology.

### Providers

Healthcare providers (eg, physicians, advanced practice providers, nurses, therapists, pharmacists, medical laboratory scientists, hospitals, clinics, and healthcare systems) are critical stakeholders in the market access process. The use of evidence-based clinical guidelines has been identified as a key factor in treatment decisions [20]. Embedded within the clinical guidelines process is a comprehensive scientific literature review. In these types of reviews, factors such as understanding the balance between benefits and harms, patient preferences, resource allocation (ie, cost and labor), feasibility, equity, and acceptability are evaluated and graded to determine the strength of the evidence and the ultimate recommendation of the clinical guidelines group [20]. Findings by Wempe and colleagues suggest that insurance coverage of an IVD affects the clinician's decision on whether to use the test [16]. Rohr et al interviewed oncologists and cardiologists about IVD use and clinical decision-making, and found that approximately half (53%) of the providers highlighted the need for clinical evidence supporting the IVD's ability to impact patient outcomes [21]. In addition, 83% of physicians highlighted the importance of combined clinical evidence to support outcomes and health economic data. Only 8% viewed economic impact as the most crucial factor in the decision-making process. IVD manufacturers should engage providers as stakeholders in a comprehensive market access strategy. For example, clinicians can provide their clinical experiences, report patient outcomes, and advocate for positive policy coverage decisions among payors. Providers can also partner with IVD manufacturers, other clinicians, and academic researchers to generate evidence to demonstrate the potential impact of a product. Findings from this type of research can then be disseminated by the external stakeholders and provide further insight into the implications for using the product. While the benefits of randomized clinical trials (RCTs) are well known, the provider can also engage in real-world evidence (RWE) generation that will evaluate diagnostic workflow, investigate clinical outcomes associated with a product, capture a broader patient population, allow evaluation of long-term clinical and patient outcomes, and assess provider insights beyond what is typically captured in an RCT [22]. Providers may also engage in studies that assess the economic impact or perform retrospective analyses of patient records to provide additional RWE that can be disseminated and incorporated into payor policy reviews [22].

### Patients

Over time, the importance of the patient perspective in the healthcare decision-making process has become clear [23]. Patients have unlimited access to healthcare information from the literature and the internet, as well as direct-to-consumer marketing for healthcare products [24]. Patients can provide insights into their experiences, help to identify unmet needs, advocate for access to new treatments, and potentially influence policy decisions [23, 25]. Stephen et al suggest incorporating the patient perspective into developing clinical guidelines [24]. By actively engaging patients in the market access process, manufacturers gain a powerful consumer voice to demonstrate effectiveness, safety, and value across the healthcare landscape [23]. Yet, beyond formal research initiatives, the best strategies for engaging patients in the market access process are not clearly or consistently defined. Patient advocacy groups (discussed later) may provide an immediate approach for engaging patients, but further research is needed to ensure relevant patient input is incorporated into the market access process.

## THE ROLE OF EVIDENCE GENERATION IN INCREASING MARKET ACCESS

Research and evidence generation are the basis for evaluating the IVD's safety, efficacy, clinical, and economic value, informing regulatory approvals, educating healthcare providers, and influencing healthcare payor coverage and reimbursement decisions [19]. The type of evidence needed may differ based on its intended purpose. Cerqueira and colleagues emphasize that evidence should ideally be generated before approvals and market introduction, when possible [26]. For example, data that IVD manufacturers submit to the US Food and Drug Administration (FDA) to demonstrate safety and effectiveness may differ from the data payors need to make coverage determinations [8]. This should be done in collaboration with industry, clinicians, public health institutions, and academic researchers. Early collaboration can aid in identifying populations of interest, selecting the appropriate research methodology, identifying data sources, and discussing potential outcomes or indications of interest relevant to all groups [26]. This fact is important because without the appropriate regulatory approvals, the IVD will not be available to healthcare providers or patients and will not be covered by payor policies. Safety, efficacy, and real-world effectiveness are primary clinical outcomes of interest to prioritize, in addition to cost-effectiveness and broader public health impact [27]. However, as previously discussed, while these approvals are essential for the product's use, they do not guarantee coverage under payor policies, as payors make their own coverage decisions.

Evidence generation in the IVD market access landscape is a multifaceted process. While studies can be led by internal IVD stakeholders, relevant evidence can also be generated by clinicians, academic researchers, and other IVD companies. The process includes collecting and analyzing data to evaluate the value proposition of a product or service focused on the healthcare environment by investigating its safety, effectiveness, cost-effectiveness, and overall cost-benefit to patients, payors, providers, and health systems [28]. Further, evidence generation of comparative clinical, humanistic, economic, and societal value of the product will be critical for coverage and reimbursement decisions, and inclusion or exclusion by clinical societies and health technology assessment organizations, which may influence payor coverage and market access [29]. The value of RWE is becoming even more critical in the market access process [28, 30]. The ability to collect data outside of the controlled environment of an RCT may add valuable data on patients’ and providers’ usage, as well as clinical outcomes. The role of clinical evidence generation is well understood for both healthcare providers and patient care outcomes. However, it is equally important to recognize the role and benefits for payors. Payors should be viewed as crucial allies in evidence generation because the types of evidence needed to inform healthcare and coverage decisions are often unavailable when a product enters the market and may not be generated as a part of an RCT. The payor can also provide critical information on the data types needed to provide product coverage or revise their policies. There is a lack of standardization and published guidelines on how payors evaluate evidence and other data for coverage determinations.

A survey of large US commercial payers revealed that while most clinical studies and guidelines were used in their evidence decisions, there were notable differences in how systematic reviews, meta-analyses, and cost-effectiveness data were used in policy coverage decisions [31]. Payor claims data are another source of evidence and can be useful for product evaluation. Claims data are generally more structured and standardized than electronic health record data, capturing information about patients regardless of where they receive care. However, they are limited by what the payor covers and may not reflect the entire healthcare experience in some cases [32]. A partnership between IVD manufacturers and payors may provide further clarity on effective strategies to translate evidence generated from claims data into payor policy decisions.

## CALL TO ACTION: MARKET ACCESS STRATEGY

Market access strategy is a comprehensive approach to ensure that the IVD is available to patients, is equitably priced, and can be reimbursed by payors [25]. The development of this strategy, along with key considerations, should begin well before the IVD receives approval. Every participant in the healthcare ecosystem can play a role in advocating for change and advancing the market access landscape for IVDs and other products (Table 2). For IVD manufacturers, the first step is developing a comprehensive market access strategy that outlines the roles of internal and external stakeholders. While some of these stakeholders are discussed in this article (eg, payors, providers, patients, regulatory agencies, clinical guideline agencies), there are many other stakeholders and roles that may be unique to the manufacturer, payor, or product. A critical aspect of the market access strategy is leveraging scientific evidence to evaluate the clinical and economic value of the product. This value proposition should be communicated to stakeholders through medical education activities, scientific journal publications, conference presentations, and engagement with healthcare providers and payors [25]. Additionally, understanding the unique needs of key stakeholders is essential. For example, a provider or payor advisory board may allow stakeholders to share insights on types of evidence used in decision-making, clinical diagnostic pathways, or the influence of clinical guidelines, which may lead to more effective market access strategies. The success of the market access strategy should be evaluated by its ability to ensure access to safe and effective products for the right patient, at the right time, in the right setting, and at the right price. The roadmap for achieving this success is multidimensional, dependent on effective partnerships with key stakeholders and the ability to influence payor coverage policies.

**Table 2.** Overview of Market Access Stakeholders

Stakeholder	Role
External market access stakeholders	
Government and regulatory agencies	Establish legislation and guidelines that govern access and healthcare budgets and make decisions about clinical efficacy and safety.
Healthcare providers	Determine treatments, diagnostic tests, and medications to prescribe for patients. Engage in evidence generation activities to evaluate impact and clinical relevance of the product.
Manufacturers	Research, develop, manufacture, demonstrate value, and complete regulatory process to bring products to market.
Patients and patient advocacy groups	Provide insights into patient needs and experiences and advocate for access to care. Engage in evidence generation activities to assess the patient and community impact of the product.
Payors and health technology assessment bodies	Assess clinical and economic value of product and determines coverage and reimbursement rates.
Independent researchers and academicians	Engage in evidence generation activities to assess the patient and community impact of the product.
Internal market access stakeholders	
Market access team	Facilitate access to products in the healthcare market, including pricing and reimbursement strategies.
Sales and marketing teams	Promotes adoption of products, communicates clinical value, and conducts market shaping activities to align products to markets.
Medical affairs teams	Provide scientific and clinical expertise, engage with healthcare providers, and implement educational initiatives about the company's products.
Senior leadership and management	Decision makers for allocation of resources, strategic partnerships, and overall direction of the company's products, which can significantly impact market access outcomes.

Adapted from: RX Communications [25].

## POLICY AND ADVOCACY

### Understanding Government Affairs, Policy, and Advocacy

Government affairs is a broad term that encompasses all aspects of how organizations interact with government entities to influence public policy decisions. At the US federal level, this includes Congress, the Judiciary, and the Executive Branch agencies. Government affairs professionals in membership societies, IVD companies, or other associations cultivate and maintain relationships with relevant agencies to advocate for their stakeholders. They use these relationships to deliver unified messages to policymakers with the aim of creating sustainable funding models that incentivize AMR-related diagnostics and therapeutics, recognizing the public health value of these technologies. Their work has led to the inclusion of diagnostics in AMR legislation, improved surveillance funding, and expanded Medicare coverage for tests [33–35]. Health policy encompasses legislative and regulatory frameworks that shape IVD development and access. Policies should promote transparency, patient safety, and innovation. However, policies may instead create administrative burdens that delay test availability. Active clinician engagement may ensure that these frameworks support timely access to diagnostics. Professional societies have also issued consensus statements advocating for increased attention to the role of IVDs in the surveillance and prevention of AMR [36–38]. When effective, these interactions foster policies guiding safe, effective IVD products to market, benefiting practitioners and patients. Having established a brief overview of government affairs, policy, and advocacy, the subsequent sections will provide examples of recent advocacy initiatives and regulatory shifts for IVDs. These examples offer practical implications of policy and advocacy within ID.

### Regulation of IVDs and Laboratory-Developed Tests

Laboratory tests are categorized as IVDs or laboratory-developed tests (LDTs) and are overseen by either CMS or the FDA. Traditionally, the FDA has served as the main interface between IVD manufacturers and the federal government, focusing primarily on enforcing regulations for commercial IVDs, largely exempting LDTs until the agency shifted its approach in 2006. In recent years, the FDA has increasingly sought to regulate LDTs, substantially altering the dynamic with IVD manufacturers, end users, and the professional organizations that represent them. Concerns have arisen over the fact that many LDTs are not fully vetted for sufficient analytic and clinical validity via current FDA and CMS regulatory oversight pathways. Starting in 2006, the FDA began drafting guidance on certain types of LDTs that it identified as lacking sufficient transparency. This guidance was not finalized, but in 2010, the FDA announced its intention to regulate all LDTs, sparking considerable debate within the public health, clinical, and diagnostic communities—a discussion that continues today [39].

Throughout the process, the FDA has provided several opportunities for stakeholder input on guidance and regulations, which is standard for the regulatory process in the US [40]. Clinicians and laboratorians provided ample feedback, expressing concern about impacts on the clinical care quality and timely diagnostic availability. Despite stakeholder feedback, clinicians and laboratorians often felt unheard by regulators. This dissatisfaction peaked when professional societies initiated a lawsuit against the FDA in response to the final LDT regulation announced in 2024, scheduled for enforcement in May 2025 [41]. Members of various clinical laboratory professional societies felt that the final regulation did not reflect their input and initiated a lawsuit against the FDA stating the agency overstepped its statutory authority in making the rule [42]. The American Society for Microbiology (ASM) supported this effort by submitting an amicus brief in support of the plaintiffs [43]. Concurrently, Congress initiated legislative efforts, highlighting advocacy's pivotal role in representing clinician and laboratory concerns. On 31 March 2025, a US District Judge ruled in favor of the plaintiffs, vacating the FDA's LDT final rule in its entirety [44]. The court concluded that the FDA's attempt to regulate LDTs as medical devices was inconsistent with current law and historical precedent. The ruling emphasized that Congress intended for CMS to oversee laboratory testing services, not the FDA.

### Policy Efforts in AMR and Antimicrobial Susceptibility Testing

The 21st Century Cures Act, signed into law in 2016, was a landmark legislation designed to accelerate medical product development and bring innovations to patients more efficiently. The Cures Act also introduced critical provisions affecting IVDs, especially in relation to antimicrobial susceptibility testing (AST) [45]. One key provision amended the federal Food, Drug, and Cosmetic Act to facilitate updating interpretive criteria—or breakpoints—for AST. Previously, these breakpoints were embedded in individual antimicrobial product labels, making timely updates cumbersome and inconsistent [45]. The FDA established the Susceptibility Test Interpretive Criteria (STIC) website in response to the Cures Act. This centralized public resource lists FDA-recognized breakpoints, including those developed by external standards organizations such as the Clinical and Laboratory Standards Institute (CLSI) [46, 47]. The availability of up-to-date breakpoints is essential to combat AMR and improve AMS efforts. The FDA's STIC website has improved transparency and consistency; however, the transition also introduced compliance challenges for IVD manufacturers and laboratories, which must demonstrate that the AST devices conform to the most current recognized breakpoints [45]. These challenges have increased in recent years as the laboratory accreditation programs—including the College of American Pathologists—now require documentation that breakpoints are current as part of their inspection checklist, creating strong motivation to prioritize timely breakpoint updates to maintain laboratory accreditation [48, 49].

ASM demonstrates strong examples of government affairs and scientific advocacy work related to AST and AMR. To address implementation gaps and ensure continued breakpoint alignment, ASM has played a central role in advocacy, coordination, and education, collaborating closely with the FDA, Centers for Disease Control and Prevention (CDC), CLSI, IVD manufacturers, clinical laboratory staff, and clinicians to support a sustainable framework for breakpoint adoption [48, 50]. ASM has also provided public comments and technical guidance to the FDA regarding the practical challenges laboratories face in updating commercial AST devices to meet the latest breakpoints [48, 50]. In parallel, ASM is actively engaging in the policy development process for Cures 2.0, a follow-up legislation aimed at building on the successes of the original Cures Act [36, 51]. Through stakeholder engagements with other professional societies (eg, Infectious Diseases Society of America), the organization has helped shape legislative proposals to ensure new policies reflect the realities of clinical microbiology laboratories and healthcare providers.

ASM has also notably led initiatives promoting antibiotic and diagnostic development incentives, including supporting legislation such as the Pioneering Antimicrobial Subscriptions to End Upsurging Resistance (PASTEUR) Act [52, 53]. The PASTEUR Act proposes novel payment models for antibiotics and IVDs critical to addressing AMR, shifting economic incentives toward sustainable market access for IVDs that guide AMS. ASM also consistently engages in advocacy emphasizing the importance of the Advanced Molecular Detection (AMD) program and the National Wastewater Surveillance System at the CDC [54, 55]. These programs highlight diagnostic capacity enhancement for emerging pathogens, including drug-resistant organisms, underlining the importance of integrated policy efforts. These various efforts—Cures, Cures 2.0, PASTEUR, AMD, and sustained engagement with the FDA, CDC, and CLSI—demonstrate how diagnostic policy can be advanced by increased collaboration between professional societies, policymakers, clinicians, and laboratorians.

## CALL TO ACTION: POLICY AND ADVOCACY STRATEGY

Clinicians and laboratorians should utilize any available opportunities to provide policymakers and regulators with feedback. It is essential for organizations and individual clinicians to comment on draft guidance, discussion papers, government and regulatory policies, and strategic plans whenever possible. Collaboration with professional associations can strengthen your influence and amplify your voice while engaging as individuals. Remember that advocacy is not limited to the federal level; involvement at state and local levels is crucial when those areas are relevant to your concerns. Industry and professional societies representing clinicians and patients can effectively promote the adoption and sustainability of IVDs for ID. This can be achieved most effectively when members are actively engaged, voicing their concerns, and offering input. It is vital to clearly communicate the significance and value of these tests—not only in terms of lives saved and improved quality of life but also from an economic perspective. One of the main challenges in effective advocacy is the lack of clear and consistent messaging. Advocates must identify the problem and clearly articulate their proposed solutions. Advocacy becomes much more difficult when policymakers encounter differing or conflicting messages.

## CONCLUSIONS

ID diagnostics are indispensable to public health, AMR/AMS, and improving patient outcomes. Despite their clinical value, regulatory and reimbursement barriers continue to limit access. As regulations evolve and legislative efforts progress, clinical and laboratory professionals must take an active role in shaping diagnostic policy (Table 3). Understanding market access, health policy, and advocacy better equips these groups to engage with payors, policymakers, regulatory agencies, and manufacturers. Through coordinated, evidence-based advocacy, stakeholders can help ensure that diagnostics are developed, reimbursed, and implemented in ways that support ongoing innovation, sustainability, and optimal patient outcomes.

**Table 3.** Call to Action Summary

Call to Action	Responsible Stakeholders	Practical Application for Stakeholders
Provide clear, consistent feedback to policymakers and regulators at all levels.	Clinicians, laboratorians, patient advocacy groups, professional societies, policymakers, and regulators.	Clinicians/laboratorians/patient advocacy groups: Identify priority public health needs for patients. Monitor proposed regulations and respond during public comment periods with clinical insights and practical feedback. Professional societies: Consolidate and amplify member input into unified policy responses. Policymakers/regulators: Proactively engage relevant stakeholders and increase transparency in the rulemaking process.
Develop a market access plan early, defining stakeholder roles and timelines.	IVD manufacturers and market access teams.	IVD manufacturers/market access teams: Conduct a comprehensive market access landscape assessment early in product development. Identify relevant internal and external stakeholders and align with regulatory and reimbursement timelines.
Leverage scientific advisory boards to gather stakeholder input and tailor market access strategies.	Clinicians, IVD manufacturers, laboratorians, and payors.	Clinicians/laboratorians: Express interest and volunteer for advisory board service. Provide perspectives for workflow and outcome prioritization. IVD manufacturers: Host advisory boards during and after product development. Leverage findings to directly inform strategy. Payors: Communicate specific data requirements and policy gaps to inform evidence planning.
Ensure patient access to safe, effective, and affordable diagnostics in appropriate settings.	Clinicians, IVD manufacturers, laboratorians, market access, patient advocacy groups, policymakers, payors, and professional societies.	All: Collaborate to raise public awareness, foster alignment, advocate for equitable reimbursement and regulatory support, and align messaging around diagnostic value. Patient advocacy groups: Represent patient voices in legislative and other policy-focused conversations and support campaigns that highlight diagnostic access barriers.
Partner with societies and industry groups to strengthen advocacy and messaging.	Clinicians, IVD manufacturers, laboratorians, patient advocacy groups, and professional societies.	Clinicians/laboratorians: Participate in joint advocacy initiatives and serve as clinical experts in public campaigns. IVD manufacturers: Support society-led efforts by supplying evidence and policy-relevant materials. Patient advocacy groups: Provide real-world examples to humanize issues and emphasize public impact. Professional societies: Lead coordinated messaging, organize advocacy efforts, and draft joint statements.
Communicate strong scientific and economic evidence through publications and outreach.	Clinicians, medical affairs, payors, professional societies, and researchers.	Clinicians/researchers: Design studies that appropriately assess the impact and value of IVDs with consideration of human behavior, implementation, and stewardship. Medical affairs: Share findings through conferences, provider education, and stakeholder briefings. Payors: Utilize published evidence to update or create new coverage policies and ensure alignment with clinical benefit. Professional societies: Publish and disseminate findings in peer-reviewed journals.

Abbreviation: IVD, in vitro diagnostic.

## Notes

***Disclaimer.*** A. K. C. and J. R. K. did not receive payment for their work on this manuscript.

***Financial support**.* Funding for this manuscript was provided by bioMérieux, Inc.

***Supplement sponsorship.*** This article appears as part of the supplement “Advancing Infectious Diseases Diagnostics Through Research, Policy, and Market Access in the US,” sponsored by bioMérieux, Inc.

***Potential conflicts of interest.*** B. K. H., A. M. P., and B. D. P. are paid employees of bioMérieux. All other authors report no potential conflicts.
